# Achiral Zeolites as Reaction Media for Chiral Photochemistry

**DOI:** 10.3390/molecules24193570

**Published:** 2019-10-02

**Authors:** Vaidhyanathan Ramamurthy

**Affiliations:** Department of Chemistry, University of Miami, Coral Gables, FL 33143, USA; murthy1@miami.edu

**Keywords:** zeolites, asymmetric photochemistry, chiral induction, organic photoreactions, solid-state photochemistry, supramolecular photochemistry

## Abstract

Obtaining enantiomerically-enriched photoproducts from achiral reactants has been a long-sought goal. The various methods developed to achieve chiral induction in photoproducts during the last fifty years still suffer from a lack of predictability, generality, and simplicity. With the current emphasis on green chemistry, obtaining enantiomerically enriched products via photochemistry is a likely viable alternative for the future. Of the various approaches developed during the last three decades, the one pioneered in the author’s laboratory involved the use of commercially-available and inexpensive achiral zeolites as the media. This approach does not use any solvent for the reaction. Examples from these studies are highlighted in this article. Since no chiral zeolites were available, when the work was initiated in the author’s laboratory, commercially-available zeolites X and Y were modified with chiral inductors so that the reaction space becomes chiral. The results obtained established the value of chirally-modified, commercial zeolites as media for achieving chiral induction in photochemical reactions. A recent report of the synthesis of a chiral zeolite is likely to stimulate zeolite-based chiral photochemistry in synthesizing enantiomerically-pure organic molecules. The availability of chiral zeolites in future is likely to energize research in this area. Our earlier observations on this topic, we believe, would be valuable for progress of the field. Keeping this in mind, I have summarized the work carried out in our laboratory on chiral photochemistry on chirally-modified zeolites. This review does not include examples where high chiral induction has been obtained via a strategy that examines molecules appended with chiral auxiliary within achiral and chirally-modified zeolites. The latter approach yields products with diastereomeric excess >80%.

## 1. Introduction

The origin and continued existence of life on earth depends on light absorption by molecules [1]. The science of light, especially in the context of organic chemistry, has a history exceeding a century. Several photochemical reactions employed today were discovered in the early 1900’s [2]. Interestingly, early photoreactions were performed in solid state [3,4]. Between 1950–70, several important photoreactions in solution, as well as in solid state, were discovered, and their mechanistic details elucidated [4]. In conjunction with discoveries of new photoreactions, the development of theoretical and physical concepts related to triplet state, radiative and radiationless transitions, and energy- and electron transfer have contributed to the vigorous growth of the field of organic photochemistry [1]. These concepts, verified by several pioneering studies from 1960–1990 with time resolved techniques, have placed the field of photochemistry on a firm footing and have allowed it to permeate other disciplines of chemistry.

In recent times, photochemistry has advanced from a purely basic science to a more applied discipline. In this progression, well-established concepts are finding use with new nomenclatures. Unfortunately, this results in the younger generation being unaware of the fundamental work done in various fields of photochemistry by the pioneers. Two such recent examples are ‘visible light photocatalysis’ [5] and ‘up-conversion’ [6], which have their origin in well investigated energy- and electron transfer processes. In the near future, similar to the aforementioned two processes, asymmetric photochemistry is likely to play an important role in the construction of complex chiral molecules. Believing a brief summary of the early contributions to this topic would be valuable and appropriate, I have outlined our work with zeolites as the media during the generation of optically-enriched products from achiral reactants. Due to space constraints, this article is limited to asymmetric photochemistry in zeolites, i.e., the work carried out in the author’s laboratory [7,8]. A number of reviews are available on this process in solution [9,10,11,12,13,14,15,16,17,18,19,20,21]. Several supramolecular assemblies have been successfully used as reaction media to achieve chiral induction [22,23]. The monograph on asymmetric photochemistry in various media by Inoue and Ramamurthy provides detailed coverage of this topic [24].

## 2. The Beginnings of Asymmetric Photochemistry

To our knowledge, the first report on chiral photochemistry in solution came from the laboratory of Hammond [25]. Hammond and Cole employed optically-active triplet sensitizers to bring about the geometric isomerization of achiral cis-diphenylcyclopropane. Although the enatiomeric excess obtained was small, this report served to seed interest in this research problem. Since that first report, several groups have performed enantio- and diastereo- selective phototransformations, both in solution [9,10] and in solid state [3]. Although several photoreactions in the crystalline state were reported in the early 1900, only in 1960s was a systematic study on this topic performed by Schmidt and coworkers [3]. Since knowledge of both crystallography and photochemistry is essential to making a significant contribution to this topic, the growth of solid-state photochemistry was slow. During the early days, the progress in asymmetric solidstate photochemistry was dependent on serendipity. Often, fortuitous crystallization of achiral molecules in chiral space groups prompted a project [26,27,28,29]. Due to this, unfortunately, relatively few examples of chiral induction during the photolysis of achiral molecules in crystalline state [30,31] were reported during this period [32]. Thus, the direct transformation of achiral molecules to optically-enriched molecules by light-induced reactions in the crystalline state has proved to be a challenge.

Slow progress, as well as a lack of clear technique to pack pure achiral molecules as chiral crystals, led to exploration into the use of chiral hosts to form chiral clathrates in the crystalline state. In this strategy, an achiral reactant molecule was enclosed within a chiral host molecule to produce a crystalline host–guest complex. The report by Natta, in which he used optically-active perhydrotriphenylene [33] as the chiral host and 1,3-dienes as the achiral guest, paved the way for future research on this topic. This approach has been elaborated upon successfully by Toda and coworkers, who employed organic chiral diol hosts [34,35,36]. Importantly, Toda and coworkers were able to achieve quantitative chiral induction in a few examples. In spite of this, no clear understanding of the rules that govern the types of molecules that would complex with the host has been reached. Therefore, this approach, while being highly promising, continues to be unpredictable. Thus, the two approaches mentioned above that are guaranteed to yield chiral products from achiral reactants are not universal, and predictability is poor.

Recognizing the need for a more general methodology, Scheffer introduced a technique known as the “ionic chiral auxiliary approach” [37,38,39,40,41]. In this method, achiral molecules are prompted to crystallize in chiral space groups by forming a chiral ionic salt from an achiral reactant (acid or base) and chiral auxiliary (base or acid). By this approach, near quantitative chiral induction has been achieved in solid-state photochemistry. Although the salt yields the products as diastereomers with respectable diastereomeric excess (de), it can be readily hydrolyzed to give the products as enantiomers (enantiomeric excess; ee). While this approach is more general, predictability is still not 100%, although there are exceptions. The main problem with photoreactions in the crystalline state is they are driven by molecular packing. However, the rules of molecular packing controlling the reactivity of molecules in the crystalline state and in solid host−guest assemblies are yet to be fully developed [42,43]. This led us to explore zeolites as media for chiral photoreactions. As outlined below, zeolites are microcrystalline solids with well-defined void spaces inside. In these spaces, organic molecules could be packed as in organic clathrates. Research from our laboratory that is briefly summarized in this review reveals that chiral induction on photoproducts could be achieved if the zeolite that is used as the reaction medium is modified with photoinert chiral inductors.

## 3. Zeolites as Media for Asymmetric Photoreactions: Chiral and Achiral Zeolites

Zeolites are inorganic microporous and microcrystalline materials that have the ability to adsorb small- and medium-sized organic molecules [44,45]. Zeolites, being porous, tend to adsorb organic molecules, both on the external and internal surfaces. For adsorption within the zeolites, the guest molecule should be able to diffuse into the interior through channels and cages that initiate on the external surface. Since the area on a zeolite’s internal surface largely exceeds that of its external surface, most adsorption takes place on the interior surface. However, the feasibility of intracrystalline adsorption depends on the kinetic diameter of the guest channels; it has to be smaller than the diameter of the passage to the intracrystalline cages and channels. The internal structure of zeolites is porous and contains well-defined cages and channels. Of the various zeolites, ZSM and faujasite types have attracted the attention of photochemists as reaction media. ZSM zeolites have narrow channels (dia: ~ 5.5 Å), while faujasites contain cages (dia: ~ 13 Å). Also, while the faujasites contain a large number of cations, ZSM zeolites have very few. The number of cations in zeolites is controlled by the framework Si/Al ratio. For chiral photochemistry, faujasites that possess larger cages and a large number of cations are valuable.

Synthetic faujasite zeolites, known as X and Y, have the following unit cell composition:

X type  M_86_ (AlO_2_)_86_ (SiO_2_)_106_ . 264 H_2_O

**Figure 1:**
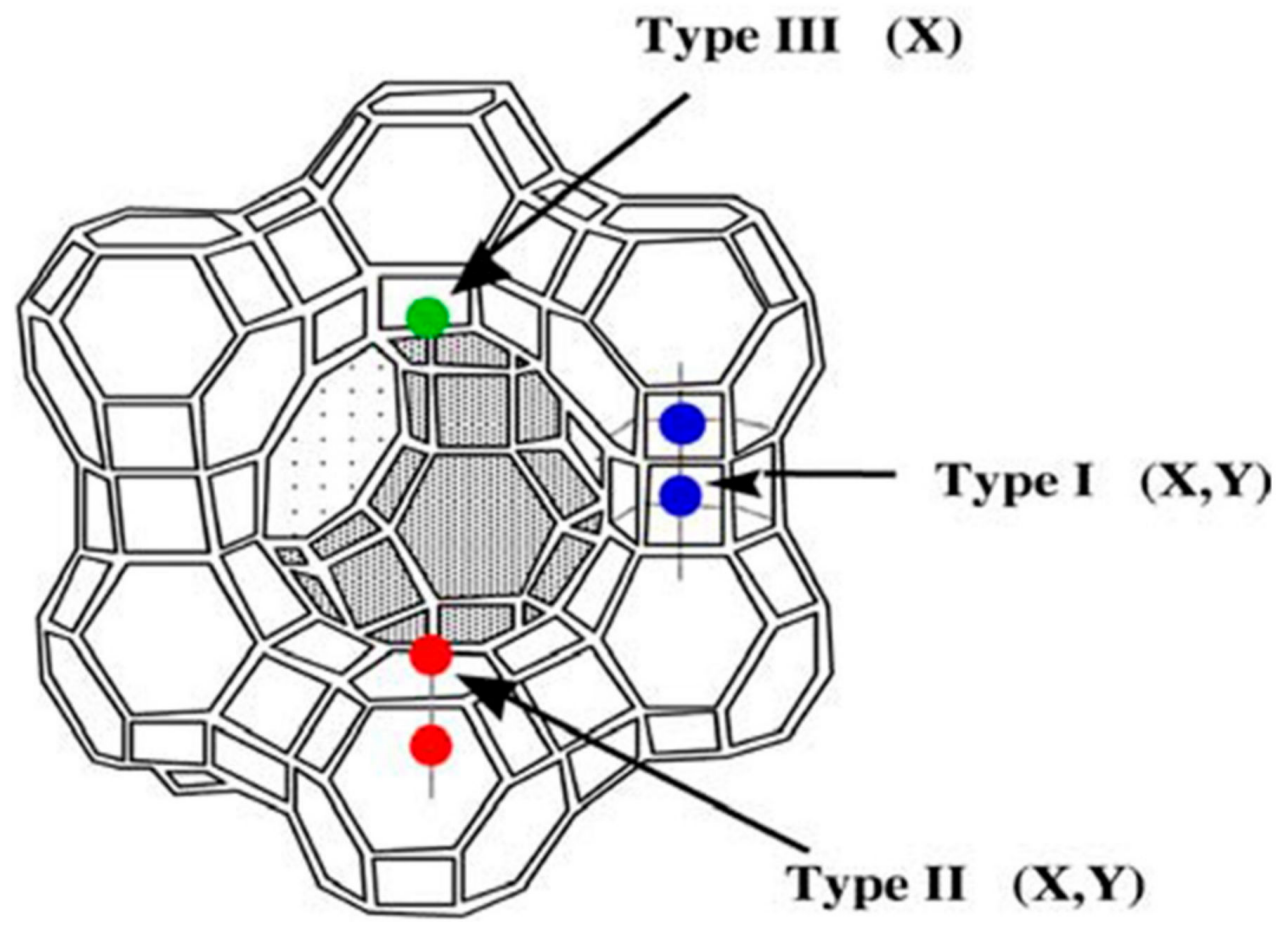
Sodalite and supercages within X and Y zeolites, and the locations of the three types of cations.

Y type  M_56_ (AlO_2_)_56_ (SiO_2_)_136_ . 253 H_2_O

where M is a monovalent cation. The ratio of AlO_2_ to SiO_2_ varies; this controls the number of cations that are associated with the framework. The internal structure of faujasites consists of two types of cages, i.e., sodalite and supercage. Smaller sodalite cages assemble to form large supercages wherein the guest organic molecules could be accommodated. Sodalite cages are too small to accommodate organic molecules. The supercages that are nearly spherical have a diameter of ~13 Å in diameter. The entry to the cage is controlled by smaller windows that are 7.5 Å in diameter. There are four windows to a cage, and they are tetrahedrally distributed about the center of the supercage. The internal porous structure of faujasites are formed by a three-dimensional network of sodalite and supercages. Since all cages are interconnected, a molecule that enters the faujasite supercage has access to all the interior cages, and can spread between multiple cages. The cations present within zeolite cages also play an important role during the photoreactions of the included guests. Since Al that replaces Si carries a negative charge, a positively-charged cation neutralizes the structure. Therefore, the number of cations present in a zeolite is equivalent to the number of Al ions present in the framework. In the context of reactions within zeolites, the cations present in the supercage play an important role. Because of sodalite cages are too small, the Type-I cations (16 cations per unit cell in both X and Y) present in sodalite cages are not important in the context of photochemistry. Organic molecules would be able to interact with Type-II (32 per unit cell in both X and Y) and Type-III cations (38 per unit cell in the case of X type and only eight per unit cell in the case of Y type) located within the supercage. Figure 1 identifies the locations of cations within X and Y zeolites.

In addition to the dimensions and the cations, the nature of the wall also influences the reaction that occurs within a supercage. In general, the cavity in which a molecule is accommodate could be classified as ‘hard’ or ‘soft’’ and ‘active’ or ‘passive’ [46,47]. Since the walls of zeolite cages are inflexible, these cages are considered hard. Because of their hardness, the walls of zeolites do not change their shape over the course of a reaction. Also, since the cations could interact with the guest molecules through weak interactions, the cages are considered to be active. Such a property would make it possible to manipulate the behavior of reactants through cations. Since the walls are inflexible, the shape and the free volume of the supercage will determine, to some extent, the nature of the product obtained from a guest molecule. The volume available for an organic molecule within a supercage depends on the number and nature of the cation. For example, it varies from 873 Å^3^ in Na X to 732 Å^3^ in Cs X).

The structural characteristics of the zeolites which have been described above make them ideal hosts to carry out photochemical reactions. Ready commercial availability at cheap prices and known easy methods to quantitatively exchange cations (both organic and inorganic) make them attractive as media with which to conduct chiral photoreactions. In order for a system to be an ideal host, it should satisfy certain criteria, namely: (i) there should be a size match between the host and the guest; (ii) the guest should possess a distinctly different electronic absorption to that of the host; (iii) the host emission, if any, should not interfere with the emission from the included guest; and (iv) included guests, upon excitation, should not undergo reaction with the host. Zeolites satisfy all the above criteria. Zeolites, like silica, scatter light and do not show emission. In spite of scattering, light can penetrate the zeolite particles and reach the molecules which are adsorbed into the interior surface. Thus, included guest molecules can be excited without any complications.

To effect chiral induction during the course of a reaction, it must occur in a chiral environment. In the case of zeolites X and Y, the reaction medium is not chiral. The importance of chiral zeolites in commercial applications have motivated several groups to attempt the synthesis of such zeolites. Success on this front has been very limited. Until a few years ago, no stable chiral zeolites had been reported. Theoretically, many can exist in chiral forms (e.g., ZSM-5 and ZSM-11). The synthesis of a few unstable chiral polymorphs of zeolite beta and titanosilicate ETS–10 have been reported [48,49]. Because of their poor stability, none of these is useful as media for chiral photoreactions. The most notable exception on this front is a report by Davis and coworkers in 1992. They were able to isolate zeolite beta enriched with polymorph A that is chiral [50]. Using this as the medium, they were able to achieve the (*R*,*R*) diol with an enantiomeric excess of 5% during the ring-opening reaction of trans-stilbene oxide. A breakthrough in the synthesis of enantiomerically-pure zeolite came after almost thirty years of initial experiments [51]. A few years ago, the Davis group reported the synthesis of a stable chiral zeolite. This publication provides a new thrust to this topic and is likely to change the value of zeolites as media for chiral photochemistry [52]. Keeping this in mind, our early work on chiral photochemistry within achiral zeolites is highlighted in this mini-review. In 1995, the author’s laboratory created an asymmetric environment within achiral zeolites by adsorbing chiral organic molecules within the supercages of X and Y zeolites [53,54]. The work described here uses achiral zeolites as the reaction media, but its internal characteristics are modified by the inclusion of a chiral inductor along with the reactant.

## 4. Achiral Zeolites Rendered Chiral

**Figure 2:**
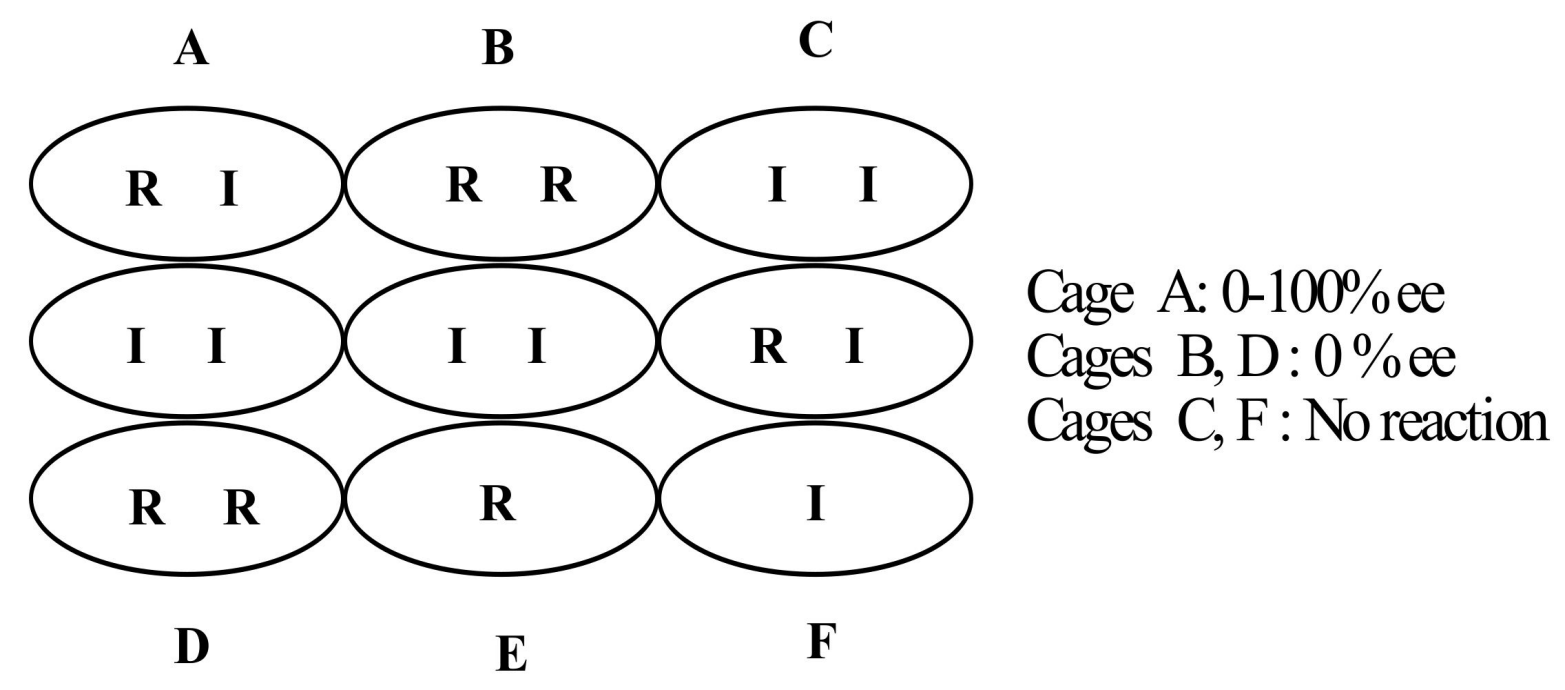
Likely distribution of chiral inductor and reactant molecules within the supercages of X and Y zeolites. Supercages are indicated as elongated oblongs. Several types of occupancies are indicated. Six types of occupancies are labeled as A–F.

In our studies, we employed three approaches to realize chiral induction in photoreactions within zeolites. The first, known as the chiral inductor methodology, involved the adsorption of a pure chiral isomer of a spectator molecule (coguest) within zeolites. By this process, the nonchiral interior space of the zeolite is rendered chiral [55]. This spectator molecule, as the name implies, does not undergo transformation during irradiation. It only provides a chiral space around the reactant molecule. The nature of the chiral inductor used to modify the zeolite surface and the extent of its interaction with the achiral reactant will determine the magnitude of enantioselectivity observed in the photoproduct. This strategy requires the adsorption of two different molecules (a chiral inductor and an achiral reactant) within the zeolite supercage (reactant (R) and chiral inductor (I)). The possible distribution of organic molecules within zeolite is represented in Figure 2: cages having two reactant molecules (*R*) (cages B and D), cages containing two chiral inductors (I) (cage C), cages containing just reactant molecule (*R*) (cage E) or chiral inductor (I) (cage F), and cages containing both reactant molecule (*R*) and chiral inductor (I) (Cage A). A similar distribution repeats within the zeolite. Since for chiral induction to occur, a reactant has to be closer to the chiral inductor, only cages containing both the reactant molecule (R) and chiral inductor (I) will yield appreciable enantioselectivity. Cages containing only the reactant molecule (cages B, D, and E) will give a product with no enantioselectivity. Hence, the observed enantioselectivity will be an average of the enantioselectivity observed from cages A, B, D, and E. The extent of enantioselectivity in cage A, as stated above, will depend on the nature of chiral inductor and its interaction with the reactant molecule which, in turn, depends on the size of the charge-compensating cations. Thus, the choice of chiral inductor and cations is critical in achieving respectable chiral induction in a photochemical reaction.

## 5. Chiral Inductor as an Active Co-guest: Photoreduction Prompted by Electron Transfer

**Scheme 1:**
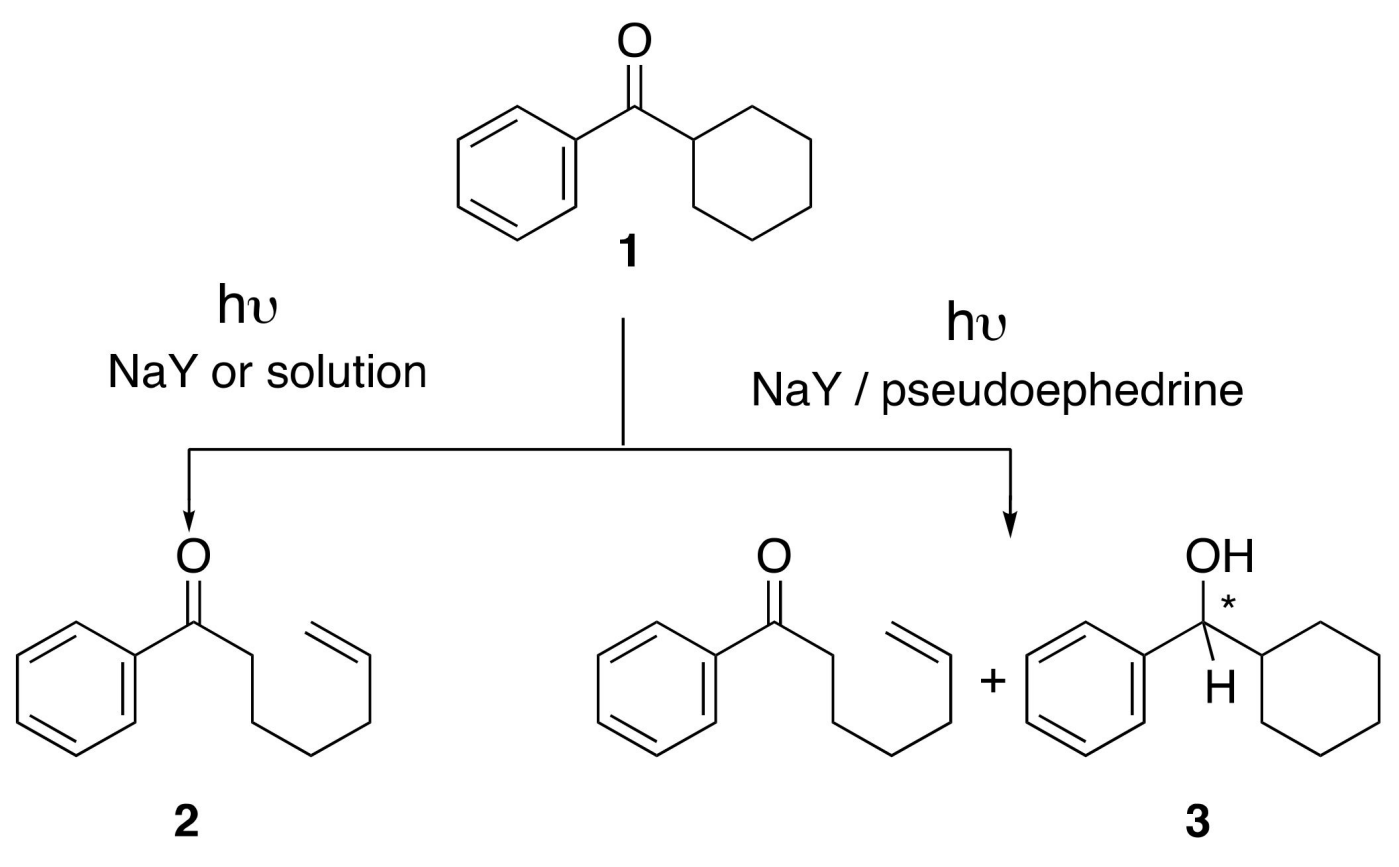
Product of electron transfer mediated reduction ανδ γ−ηψδρογεν abstraction of phenyl-cyclohexyl ketone.

**Scheme 2:**
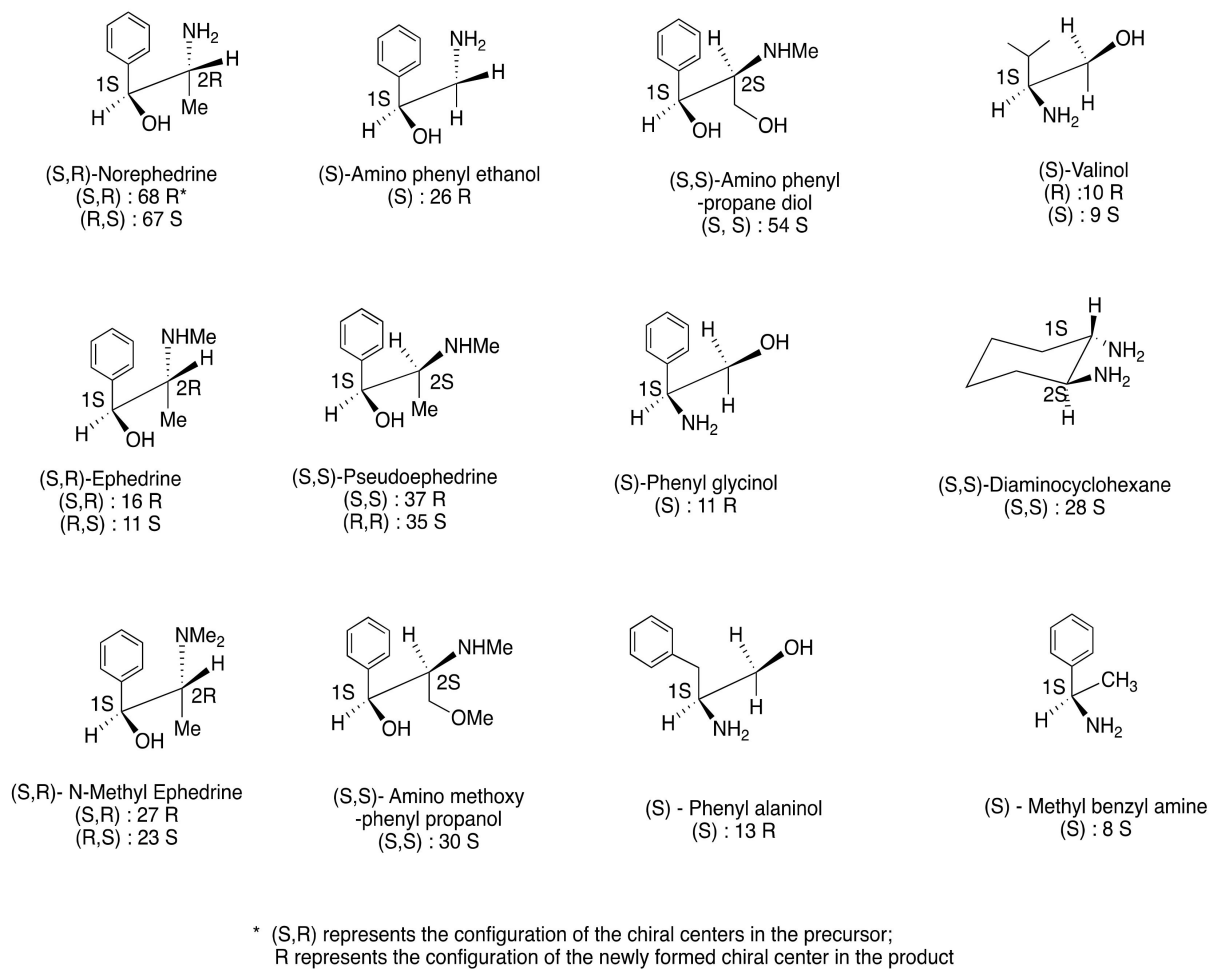
Enantiomeric excess (ee) obtained in the case of product 3 from 1 with various chiral inductors within NaY zeolite.

**Scheme 3:**
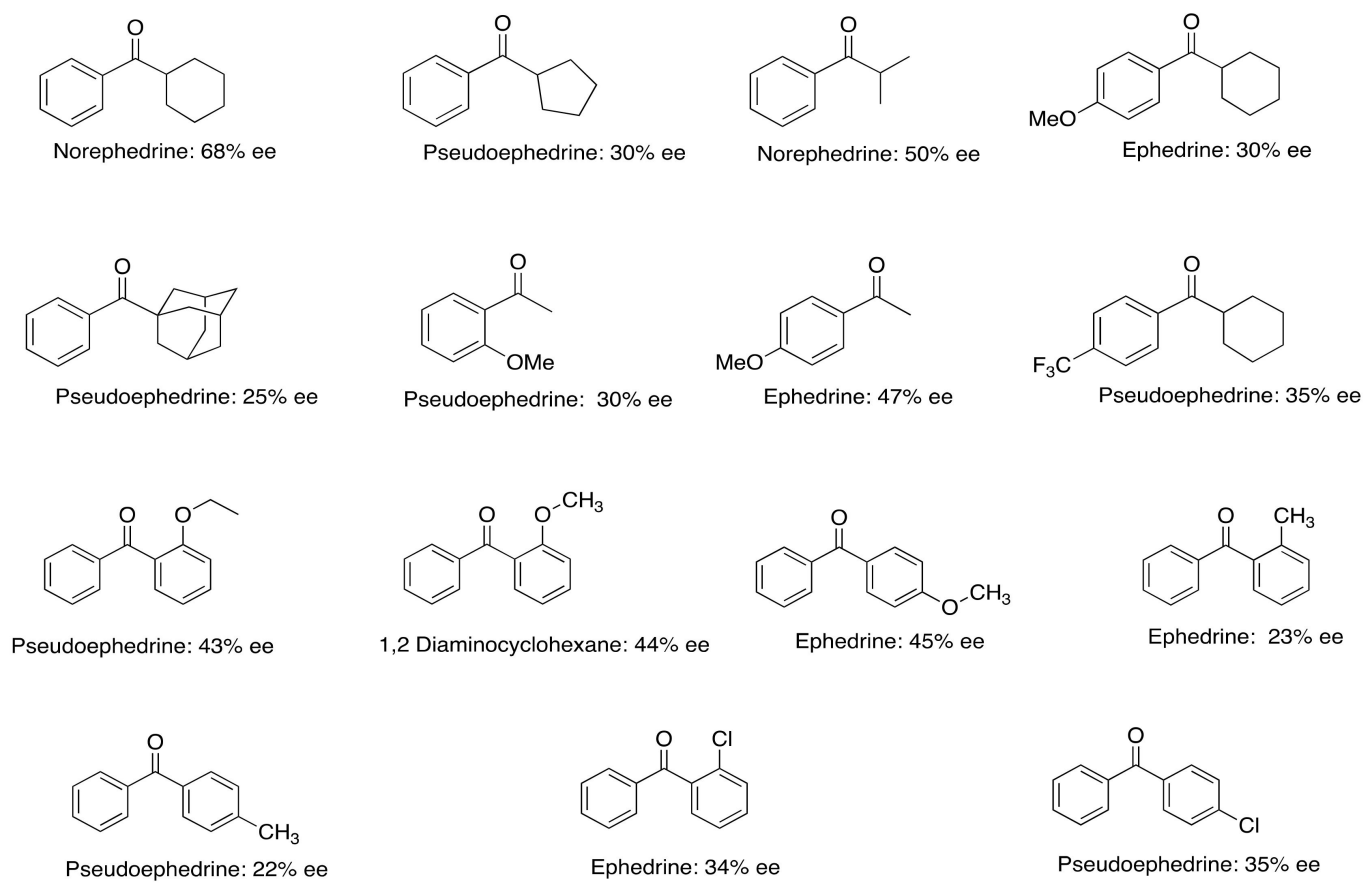
Enantiomeric excess (ee) obtained in the case of various arylalkyl ketones within NaY zeolite with the chiral inductor giving the best ee’s.

In the approach described above, there is very little control over the distribution of the reactant and the chiral inductor. It is obvious, without a clear understanding of the factors that control the distribution (Figure 2), that it is hard to predict the outcome of the chiral induction in this medium. Since the goal in the initial stages of this study was to establish the feasibility of using achiral commercial zeolites as media for chiral induction, a reaction that would occur only if the reactant is next to a chiral inductor was chosen as the probe reaction. This choice restricts the reaction to cage A in Figure 2. To fulfill this criterion, we chose an amine-induced photoreduction of carbonyl compounds (Scheme 1) [56,57,58]. This reaction is prompted by an electron transfer from amine to an excited ketone. If the amine is a chiral amine, it can also serve as a chiral inductor, in addition to being an electron donor. Carbonyl radical anions generated by electron transfer would abstract a proton and yield an optically-active alcohol [59]. Normally in the absence of a chiral bias, a racemic mixture of alcohol would be produced. However, when the generated carbonyl radical anion intermediate is adjacent to a chiral inductor, there is a good possibility of a preference for the abstraction of a proton from one of the two pro-chiral faces of the ketone. To test this hypothesis, we examined the photoreduction of phenyl cyclohexyl ketone **1** that normally undergoes an intramolecular, Type-II reaction in solution. However, in the presence of an electron transfer agent, it could undergo photoreduction to give **3**. In contrast to isotropic solution, within a zeolite in the presence of chiral amines such as ephedrine, pseudoephedrine, or norephedrine intermolecular reduction product, α-cyclohexyl benzyl alcohol **3** was the main product (Scheme 1). Chiral amine is indeed the electron transfer agent, as was revealed by the dependence of the ratio of intermolecular reduction product vs. Type-II product (**3**/**2**) on the nature of the amine (primary, secondary or tertiary). Amongst the various amines used, tertiary amine functioned better as the electron donor, and yielded larger amounts of the intermolecular reduction product. The ee obtained with various amines are listed in Scheme 2. Amongst the various chiral inductors listed, norephedrine, possessing a primary amine functionality, worked better as a chiral inductor (ee 68%). Norephedrine is the source of chiral induction, as confirmed by using the antipode of norephedrine (SR vs. RS). It is important to note that under similar conditions, in solution, no chiral induction was obtained in the reduction product. The generality of this chiral induction within zeolite was established with a number of aryl alkyl and diaryl ketones (15 in total; Scheme 3), as well as chiral inductors (Scheme 2). This example served as the proof of principle of the viability of chiral induction within achiral zeolites. However, the % ee was not high, and the overall process was not synthetically useful. Yet, this is the first example of the chiral induction of a photoreaction within a zeolite, and also possibly a photoreaction with high chiral induction.

## 6. Chiral Inductor as a Passive Co-guest: Chiral Induction on Photoproducts within Zeolites

In the method described in Section 5, the chiral inductor initiated the reduction process by getting involved in electron transfer. This required the chiral inductor and the reactant to interact closely. In this section, we provide examples where the chiral inductor acts only as the chiral inductor, and does not get involved in the reaction at any stage. Thus, there is only physical interaction between the chiral inductor and the guest; there is no chemical interaction. In these examples, the confinement provided by the zeolite medium and the weak interaction between the charge-compensating cations and the guest forces an interaction between the achiral reactant and the chiral inductor. The moderate optical induction obtained in these examples shows that even achiral zeolites modified with chiral inductors could serve as a valuable reaction media for photoreactions that involve the transformation of achiral reactants to chiral products. The examples provided below not only illustrate that this approach works well with selected systems, but also provides encouragement for future studies on this topic.

### 6.1. Photocyclization of Tropolones

**Scheme 4:**
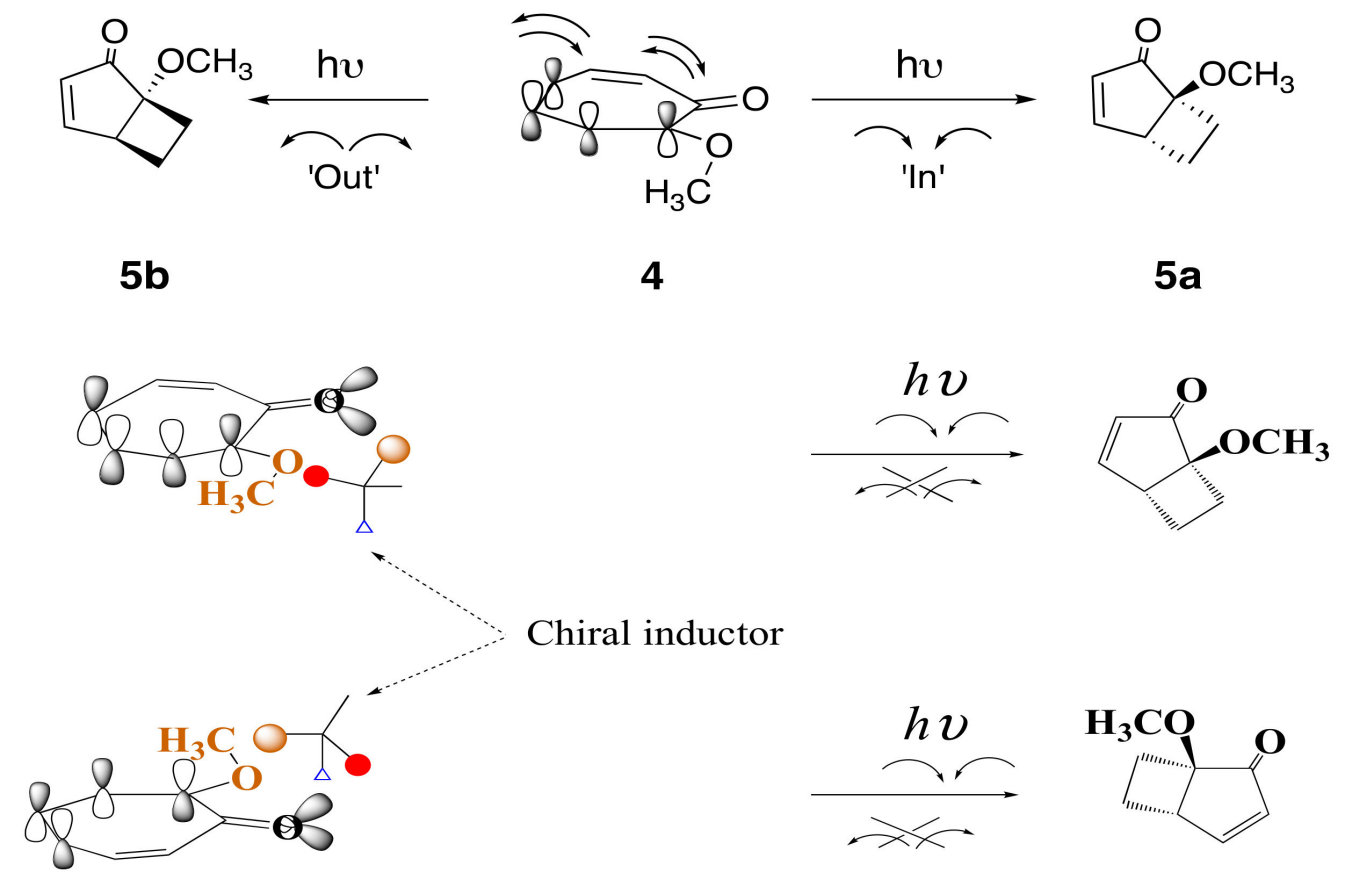
Top: Photocyclization of tropolone alkyl ethers to yield chiral products. Bottom: The chiral inductor guides the tropolone to adsorb from one prochiral face. Note that the steric interactions are different during adsorption through different enantiotopic faces. The three groups are illustrated with different shapes and colors.

Tropolone alkyl ether (**4**), upon excitation, undergoes 4e ‘dis’ rotatory ring closure. As shown in Scheme 4, opposite optical isomers are obtained upon disrotation, that could occur in two different ways (**5a** and **5b**). When the molecule is adsorbed on the surface of a zeolite, one mode of rotation will be restricted, leaving the other to dominate; in principle, this could lead to chiral induction. However, since tropolone could adsorb through both enantiotopic faces of the molecule, even if one mode of rotation is restricted, equal amounts of the two enantiomers of the products would be obtained by rotation through one mode of rotation from both enantiotopic faces (Scheme 4). Thus, the adsorption on a surface alone is not expected to bring about enantioselectivity. Most important is to encourage a tropolone molecule to adsorb on the zeolite surface through only one pro-chiral surface. This is likely if the surface is artificially altered to be chiral. This was achieved by adsorbing optically-pure chiral molecules within achiral zeolites. In Scheme 4, the means by which a chiral inductor present on a surface may direct the mode of adsorption by tropolone alkyl ether is illustrated. A surface that can hold the chiral inductor as well as the reactant tropolone alkyl ether firmly in only one fashion is needed to achieve the desired goal. In this context, zeolitic cations are expected to strongly interact with chiral inductors, and thus present them in certain geometries to the reactant molecule [60,61].

**Scheme 5:**
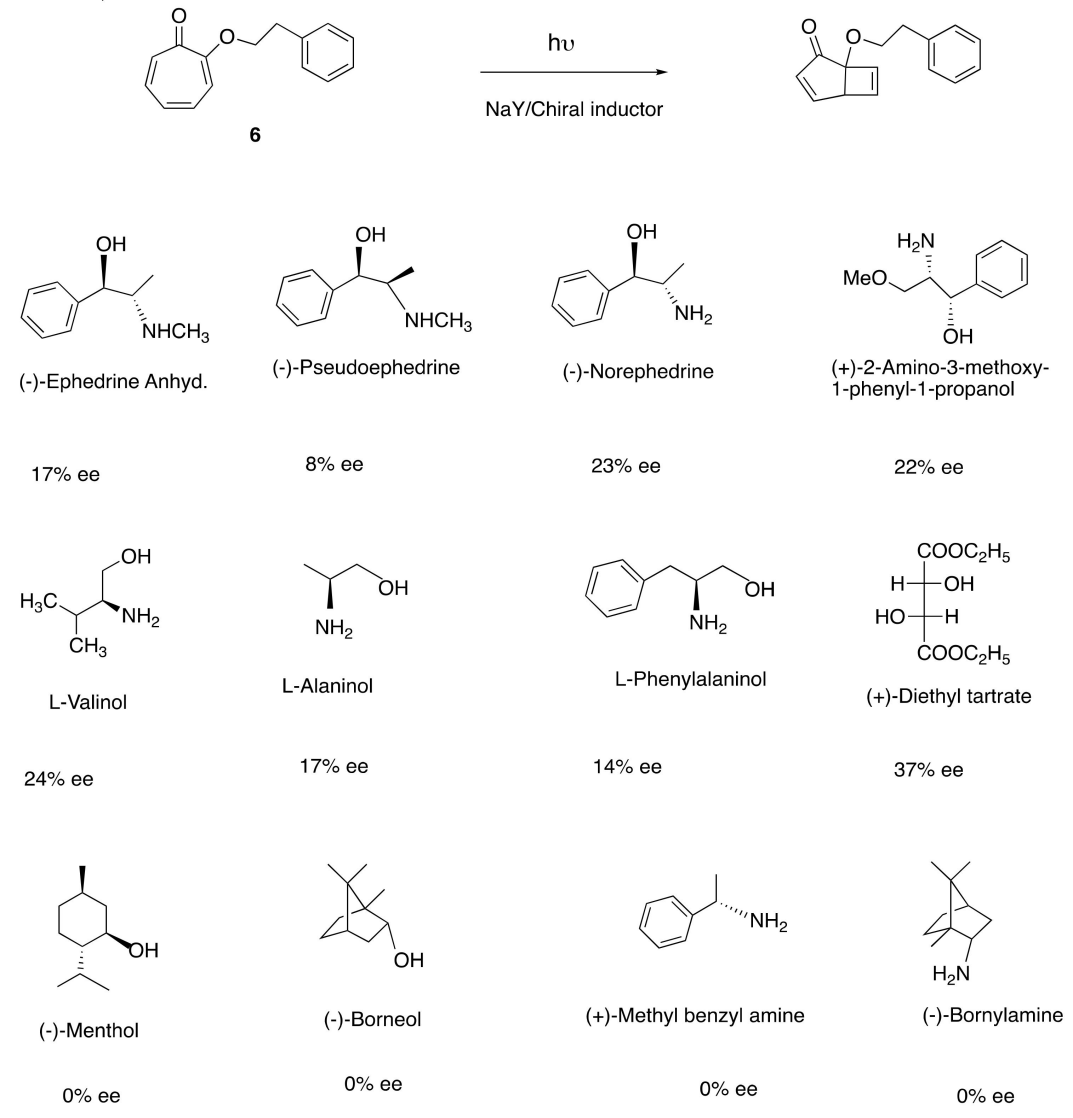
Enantiomeric excess obtained with various chiral inductors during photocyclization of tropolone alkyl ether within NaY zeolite [63].

**Scheme 6:**
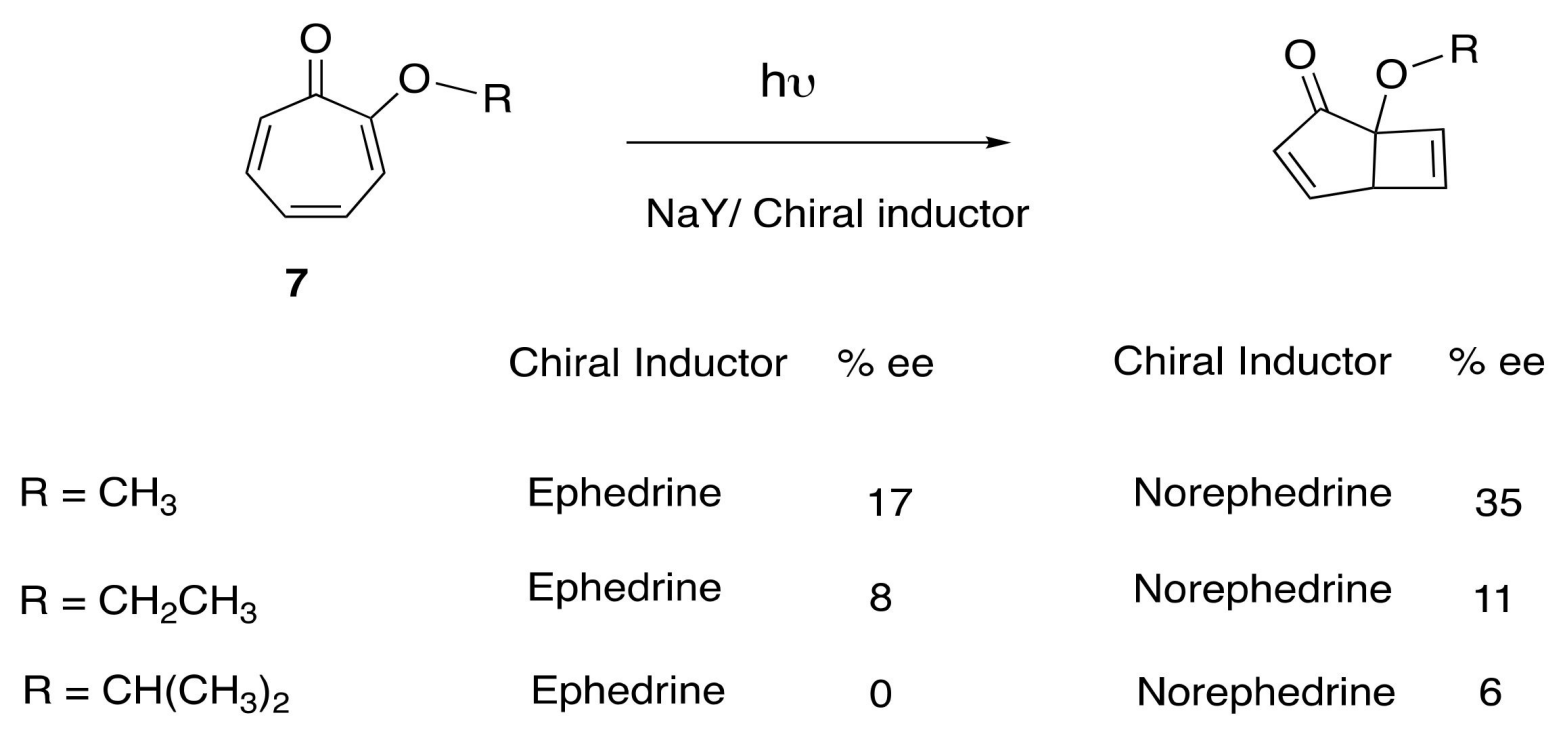
Photocyclization of tropolone alkyl ether of different chain length within NaY zeolite [65].

**Scheme 7:**
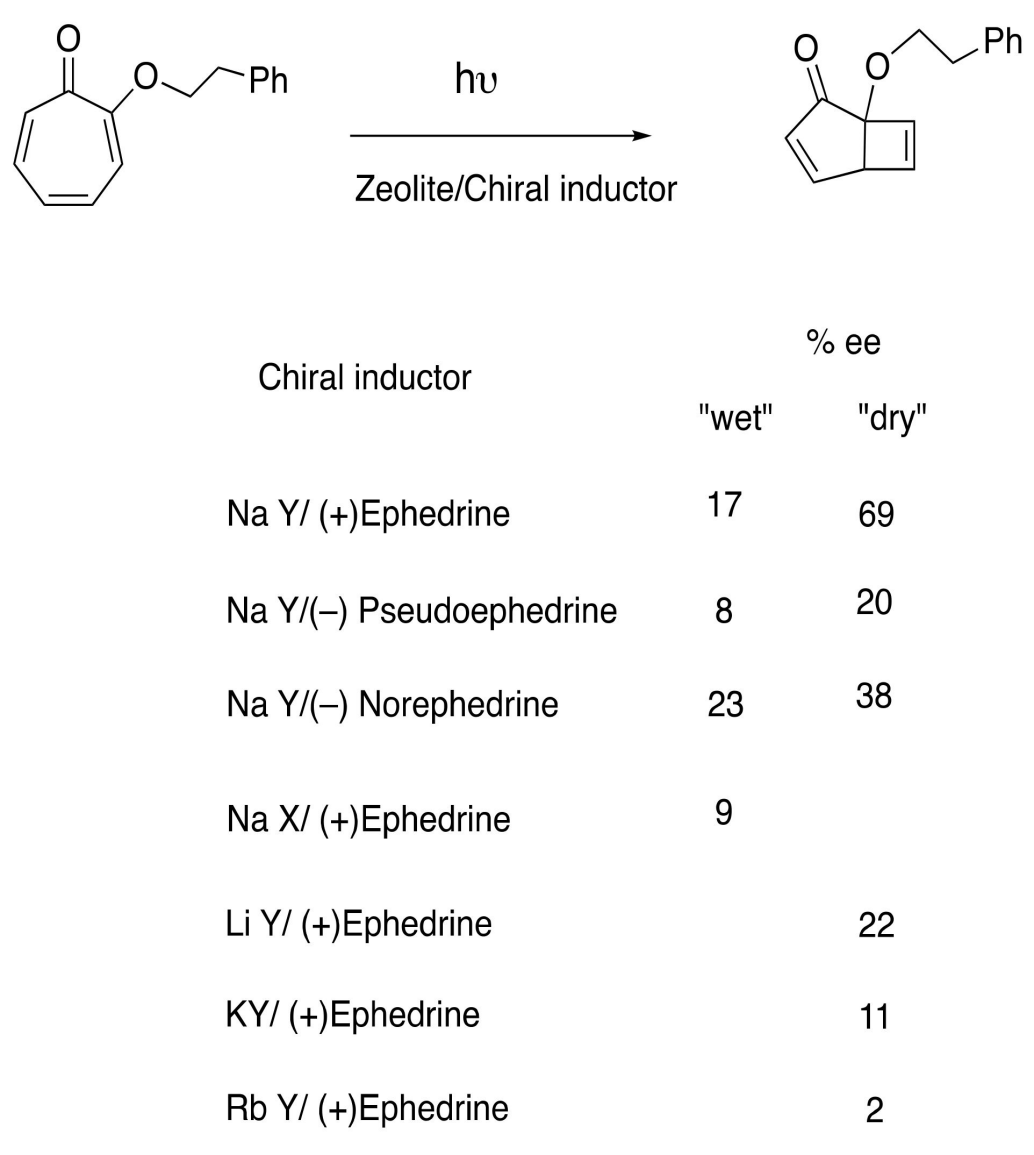
Photocyclization of tropolone alkyl ether within MY zeolite. Note the change in ee with the cation of the Y zeolite, with water content and with the variation of the chiral inductor [65].

**Figure 3:**
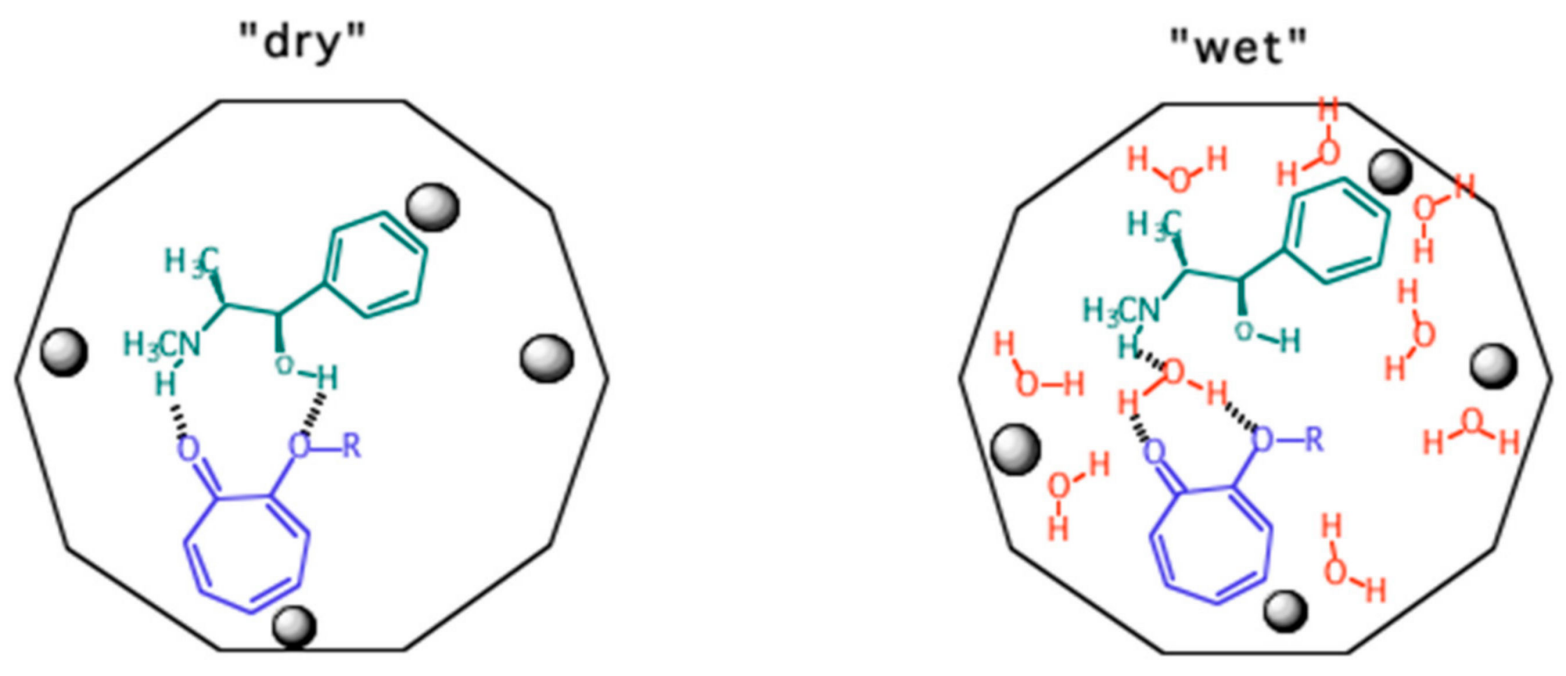
Suggested orientation of the chiral inductor and the reactant within dry and wet zeolites. In dry zeolites, the cation is free, while in wet zeolites, they are surrounded by water molecules [65].

The results presented in Scheme 5, Scheme 6 and Scheme 7 show that electrocyclization can proceed in a stereoselective fashion within zeolites [62,63,64,65]. Maximum ee in the case of **6** and **7** (Scheme 5) was obtained with bifunctional chiral agents, suggesting that multipoint interaction is essential for chiral induction to occur within zeolites. This suggested the need to identify bifunctional chiral inductors to modify the interior of zeolites. It is important to note that the extent of chiral induction depends on the nature of cations (Scheme 7). [65] Apparently, weak interactions between the cation, the chiral inductor, and reactant play a crucial role in the chiral induction process within zeolites. This is also evident from the results observed with wet and dry zeolites. [63] The inability of hydrated cations to anchor the tropolone alkyl ether to the zeolite surface is reflected in the decreased ee. Figure 3 provides a model that should aid one to visualize the mechanism of chiral induction within zeolites [63]. The recognition points, in most cases, are likely the hydroxyl, amino, and aryl groups of the inductor, the cations of the zeolite, and the carbonyl and methoxy groups of tropolone alkyl ether. The fact that the extent of chiral induction (% e.e.) and the direction (i.e., which isomer is enhanced) depend on the nature of the zeolite, X vs. Y (X and Y differ only in terms of the number of cations within a supercage), and the cation suggests that the presence of smaller cations like Li^+^, Na^+^, and K^+^, and the absence of water molecules are essential to the chiral induction process (Scheme 7).

### 6.2. Photocyclization of Pyridones

**Scheme 8:**
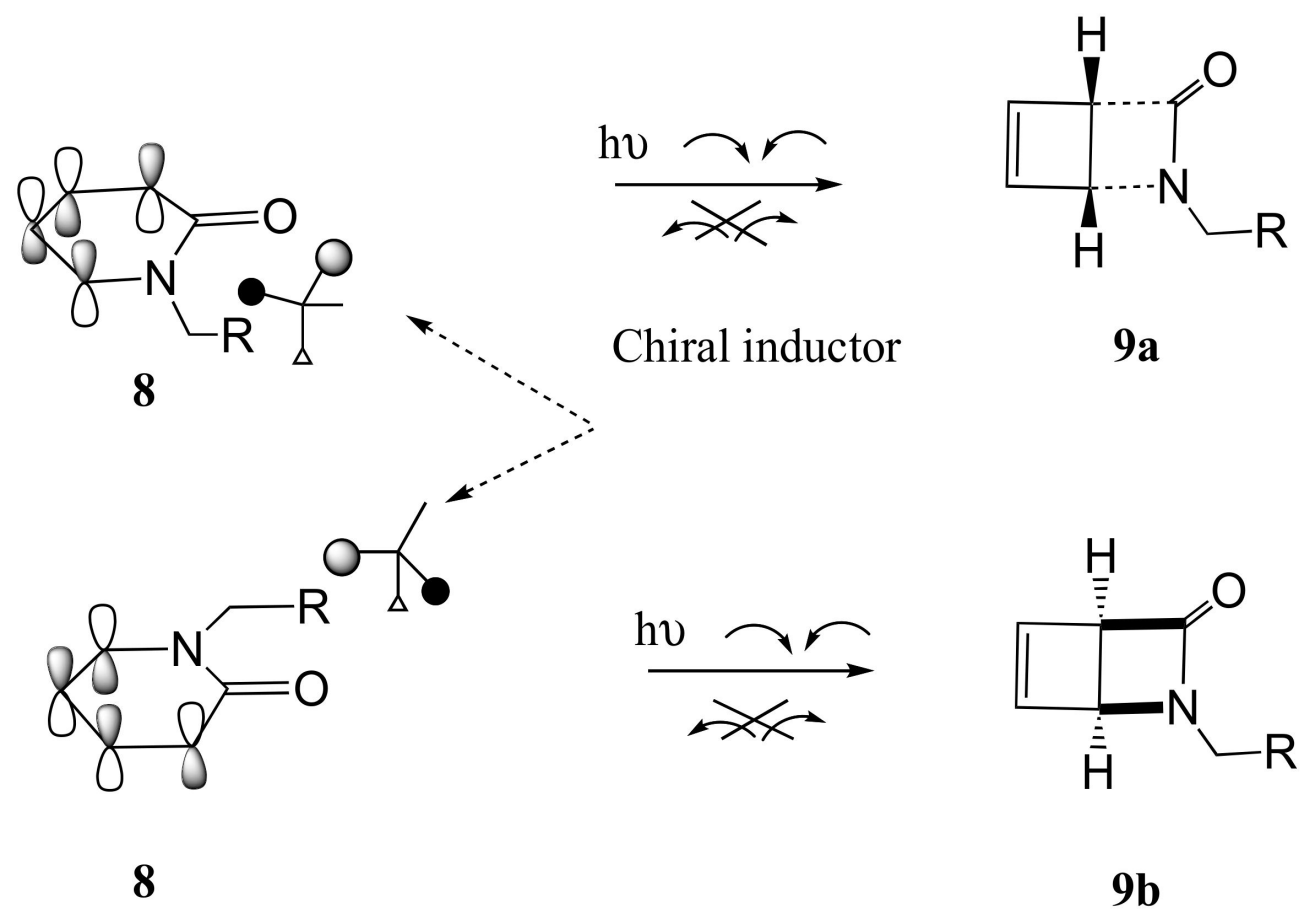
Photocyclization of *N*-alkyl pyridones. Two modes of electrocyclization would yield enantiomeric products. The adsorption of the reactant pyrione on a surface facilitated by a chiral inductor could lead to enantiomeric excess in the product.

**Scheme 9:**
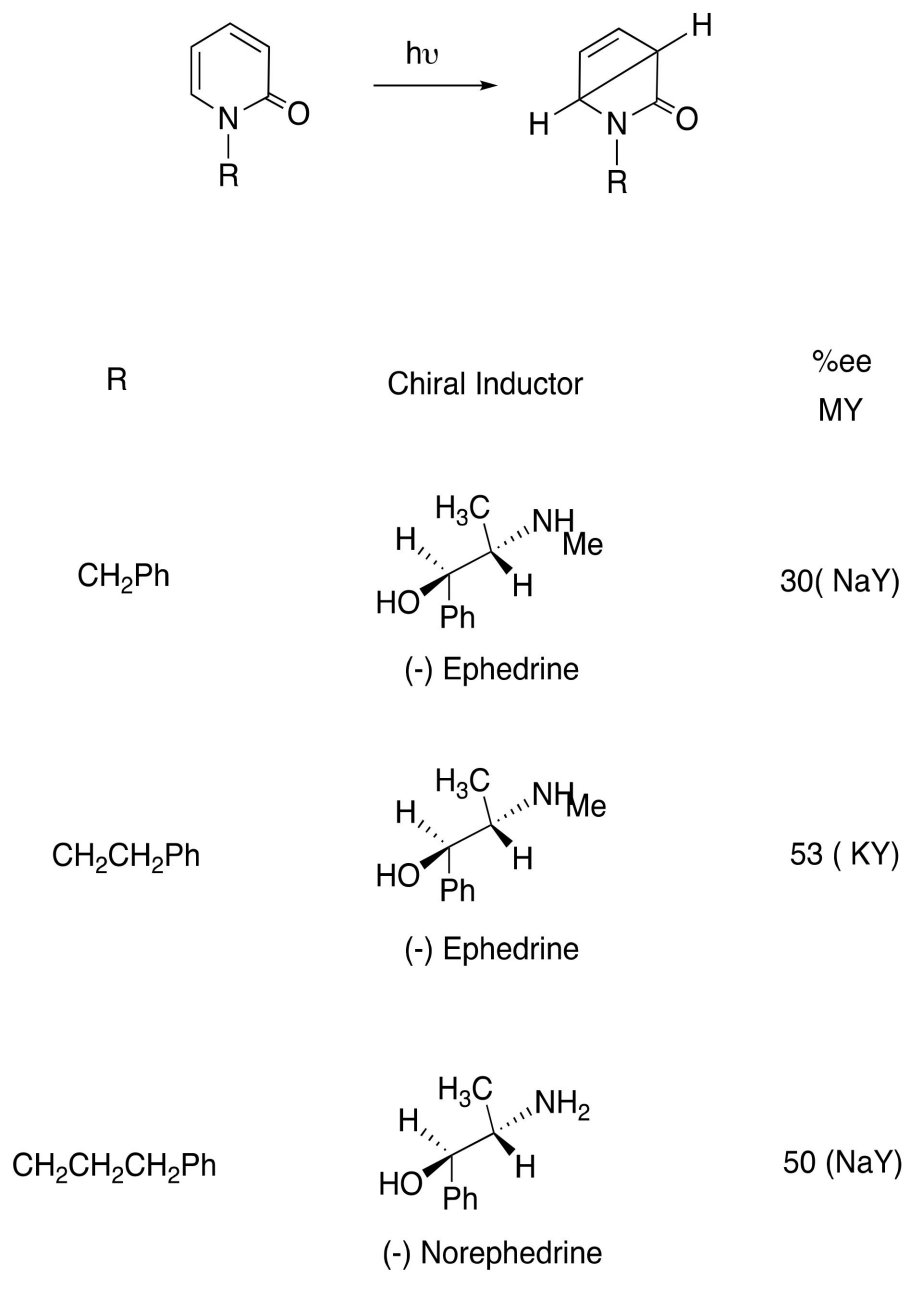
Photocyclization of *N*-alkyl pyridones and chiral induction in the product within various Y zeolites. The best chiral inductor and the best zeolite are indicated.

The photobehavior of *N*-alkylpyridones (**8**) within zeolites provides further support to the claim that chirally-modified zeolite is a very useful medium to obtain chirally-enriched products from achiral reactants (Scheme 8). Achiral pyridones, upon irradiation, undergo intramolecular 4π disrotatory photocyclization, similar to tropolones, to yield chiral β-lactams (**9a** and **9b**). As in the case of tropolones, controlling the direction of photochemical ring closure should result in asymmetric induction in the photoproduct. Various methodologies have been reported in the literature for conducting asymmetric photocyclization of pyridones. The maximum enantioselectivity (~20 %ee) in solution was achieved by Bach et. al. in the presence of a chiral host [66]. The author’s group succeeded in employing a chirally-modified zeolite as a medium to achieve chirally-enriched product from pyridones via photocyclization [67]. Of the three examples listed in Scheme 9, two yield cyclized products with moderate enantioselectivity (ee > 50%). 

In isotropic media, there will be an equal opportunity for cyclization from both tropolones and pyridones. To obtain stereoselectivity, it is necessary to exert control over the mode of cyclization. Similar to that in the case of tropolones, the chiral inductor within a zeolite helps the pyridone molecule to adsorb on the surface from one of the two enantiotopic faces (Scheme 4 and Scheme 8). The fact that the reaction, when performed within zeolite without a chiral inductor gives a racemic mixture, suggests the important role played by chiral inductors in achieving moderate enantioselectivity. Even though, the cation and the zeolite framework help to control the observed stereoselectivity, and the stereoselectivity is not quantitative, stressing that more needs to be understood about the zeolite-based chiral inductor strategy.

### 6.3. Photoisomerization of 1, 2-Diphenylcyclopropanes

**Scheme 10:**
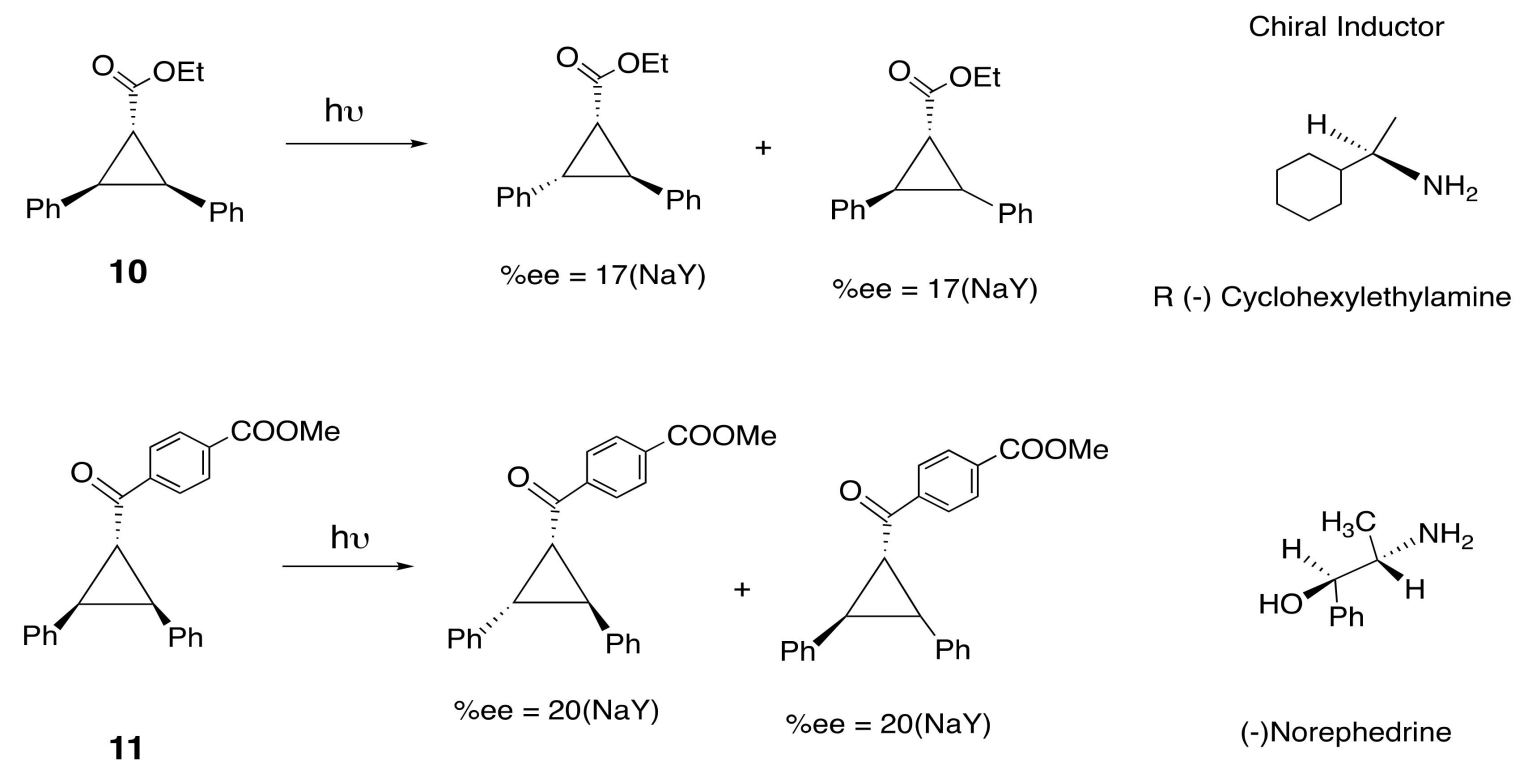
Photoisomerization of achiral cis-1,2-diphenylcyclopropane leads to chiral trans-1,2-diphenylcyclopropane. Within chirally-modified zeolite, the isomerization could be stereoselective.

As outlined in Section 2, the photoisomerization of 1,2-diphenylcyclopropane is the first photoreaction that showed promise for the feasibility of chiral induction in solution [25]. Cis-1,2-diphenylcyclopropane is optically-inactive due to the presence of a plane of symmetry in the molecule. However, since the plane of symmetry is compromised in trans-1,2-diphenylcyclopropane, it is optically active. The photoisomerization of optically-inactive cis-1,2-diphenylcyclopropane to its optically-active trans from could be brought about by both direct and triplet-sensitized irradiations and electron transfer sensitization [25,68,69]. In the examples discussed in this section, the chiral inductor that induces enantioselectivity is adsorbed on the zeolite interior surface to provide a ‘local chiral environment’, and is not linked to the reactant through either covalent or ionic bonds. Asymmetric induction ensues as a result of the close proximity between the reactant and the chiral inductor within the confined space of the zeolite supercage. For this method to work effectively, the chiral inductor must interact with the reactant and the cation via non-covalent bonding. In this context, the photoisomerization of 2,3-diphenylcyclopropane-1-carboxylic acid derivatives (**10** and **11**) within zeolites was investigated (Scheme 10). In the two reactions listed in Scheme 10, low but significant ee was obtained with several chiral inductors [70,71,72]. For example, ethyl ester undergoing photoisomerization in an isotropic medium yielding a racemic mixture of the corresponding trans-isomer gave the same isomer in 17% ee within chirally-modified NaY, with optically-pure cyclohexylethylamine. The use of the optical antipode of the chiral inductor gave the opposite enantiomer to the same extent as expected, indicating that the system is well behaved inside the zeolite. It is encouraging to note the fact that, unlike solution, chirally-modified zeolite was able to bring about some degree of chiral discrimination. Further work is needed to understand the reasons for the low ee. It may be of interest to note that these systems present high diastereomeric excess by the chiral auxiliary strategy within zeolites.

### 6.4. Oxa-di-π-Methane Rearrangement of Cyclohexadienones

**Scheme 11:**
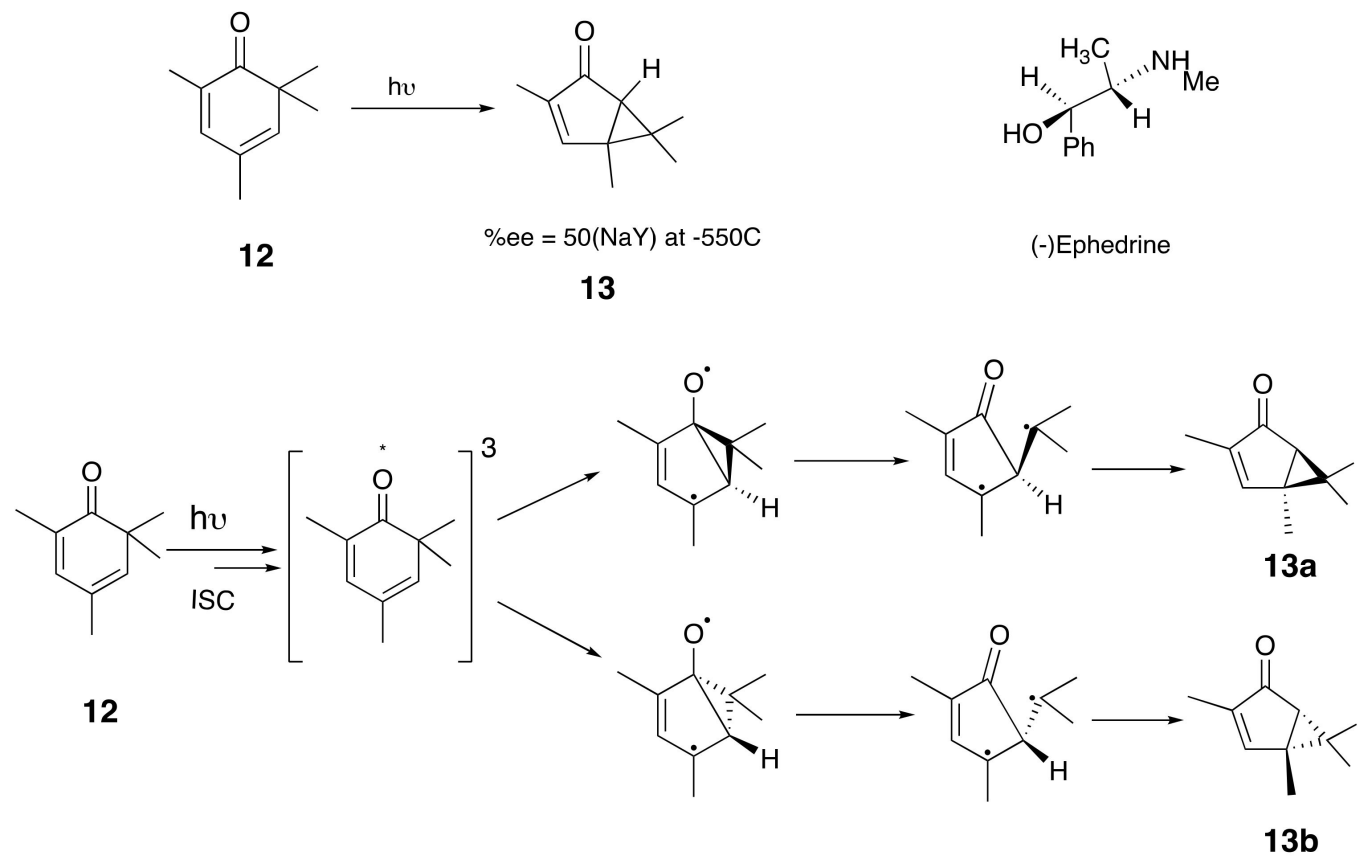
Products of photorearrangments (geometric isomerization and oxa-di-π-methane rearrangements and ee obtained within NaY in presence of a chiral inductor.

As illustrated in Scheme 11, 6,6-dimethyl-2,4-cyclohexadienone (**12**) undergoes oxa-di-π methane rearrangement to give a bicyclic product. In this reaction, the chirality is induced into the product during the first step of the reaction. In the species where radicals are centered at the oxygen and the tertiary carbon, formed from the triplet of the reactant, the cyclopropane ring can either be above or below the plane of paper. Similar to tropolone and pyridone discussed above, the cyclic dienones do not bind to one particular face preferentially to the zeolite cavities, and so in the absence any external chiral reagent, the photoreaction yields racemic products. This is also the case in solution. The irradiation of **12** included in zeolite NaY (hexane slurry) gave the oxa-di-π-methane rearranged isomer as the sole product, with equal amounts of both enantiomers (**13a** and **13b**). In solution, even in the presence of a chiral inductor, ephedrine, a racemic product mixture resulted. However, the irradiation of a hexane slurry of the above compound included in dry (−)-ephedrine-modified NaY made the product enantiomerically enriched to the extent of 30 ± 3% [67,72,73]. As expected, the optical antipode (+)-ephedrine gave the opposite enantiomer in 28 ± 3% excess. Among the various chiral inductors examined, pseudoephedrine gave respectable ee ((+) isomer 26% and (−) isomer 24%), whereas all others (menthol, valinol, methylbenzylamine, norephedrine, and diethyltartrate) yielded the product in less than 20% ee. Variation of the irradiation temperature had a distinct effect on ee: with (−)-ephedrine as chiral inductor, the ee, at −55 °C was 49%, while at 100 °C, it was 7%.

### 6.5. Norrish-Yang Photocyclizations

**Scheme 12:**
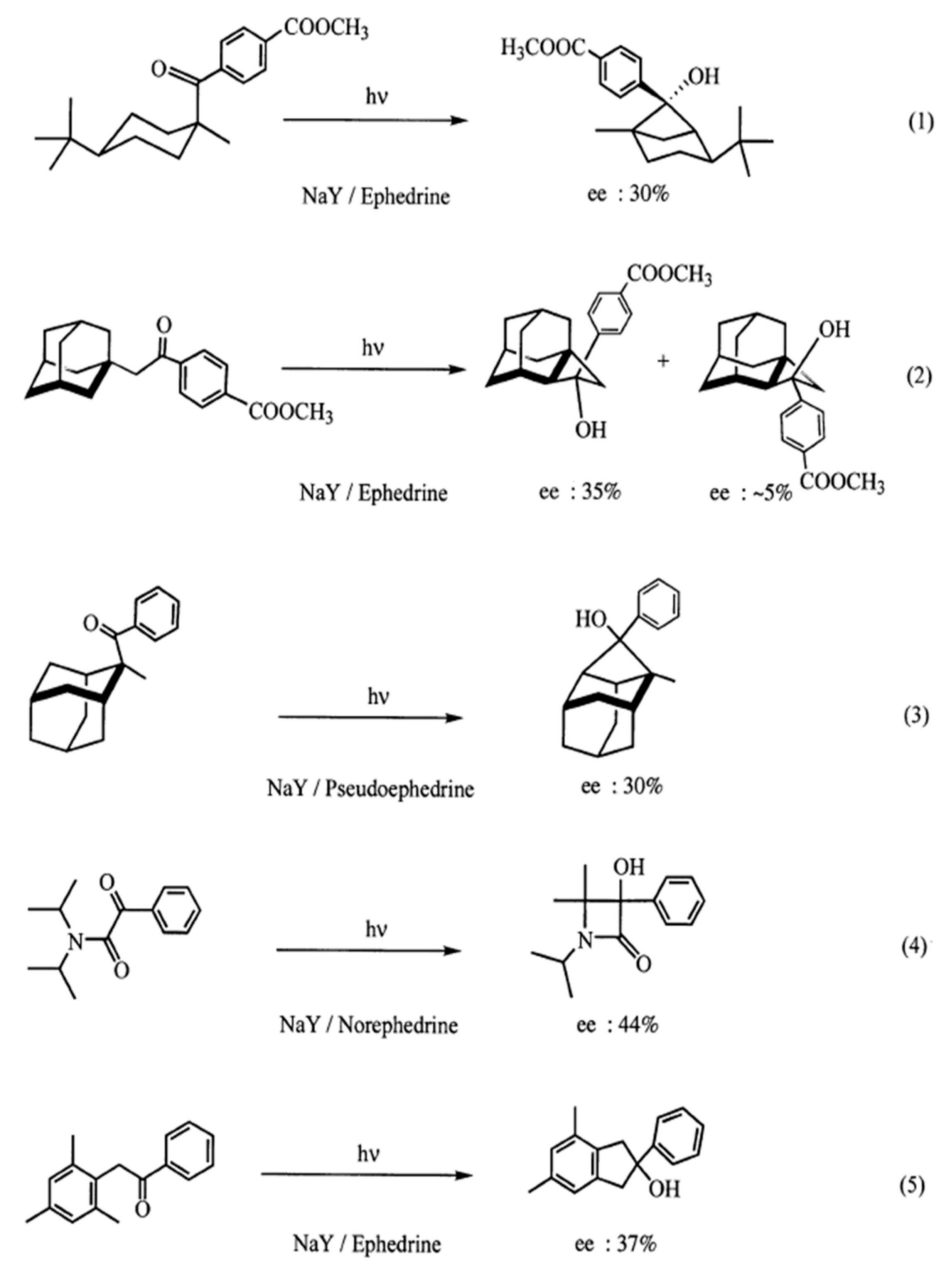
Intramolecular γ-hydrogen abstraction (Norrish-Yang cyclisation) reactions of arylcycloalkyl ketones.

The generality of chiral induction within chirally-modified zeolites was tested with the classic Norrish Type-II γ-hydrogen abstraction reaction. In this reaction the reactant is achiral and the Yang product (cyclobutanol) is chiral. Examples of this reaction are provided in Scheme 12. In all cases, the products in solution (even in the presence of chiral inductors) are racemic. Enantiomeric excess in the range of 30% is routine within chirally-modified NaY modified zeolites [60,74,75]. The examples presented in this section show that chiral inductors function better within a zeolite than in solution.

## 7. Summary

In this review, we have highlighted chiral induction in a variety of photoreactions of achiral molecules within achiral zeolites with the help of chiral inductors. The approach outlined here has employed readily-available and inexpensive zeolites for this purpose. The achiral nature of the commercially-available zeolites was overcome by modifying them with chiral inductors. The examples demonstrate that such chirally-modified zeolites could serve as a chiral medium to achieve low-to-moderate enantiomeric excess in photochemical reactions [55]. 

**Figure 4:**
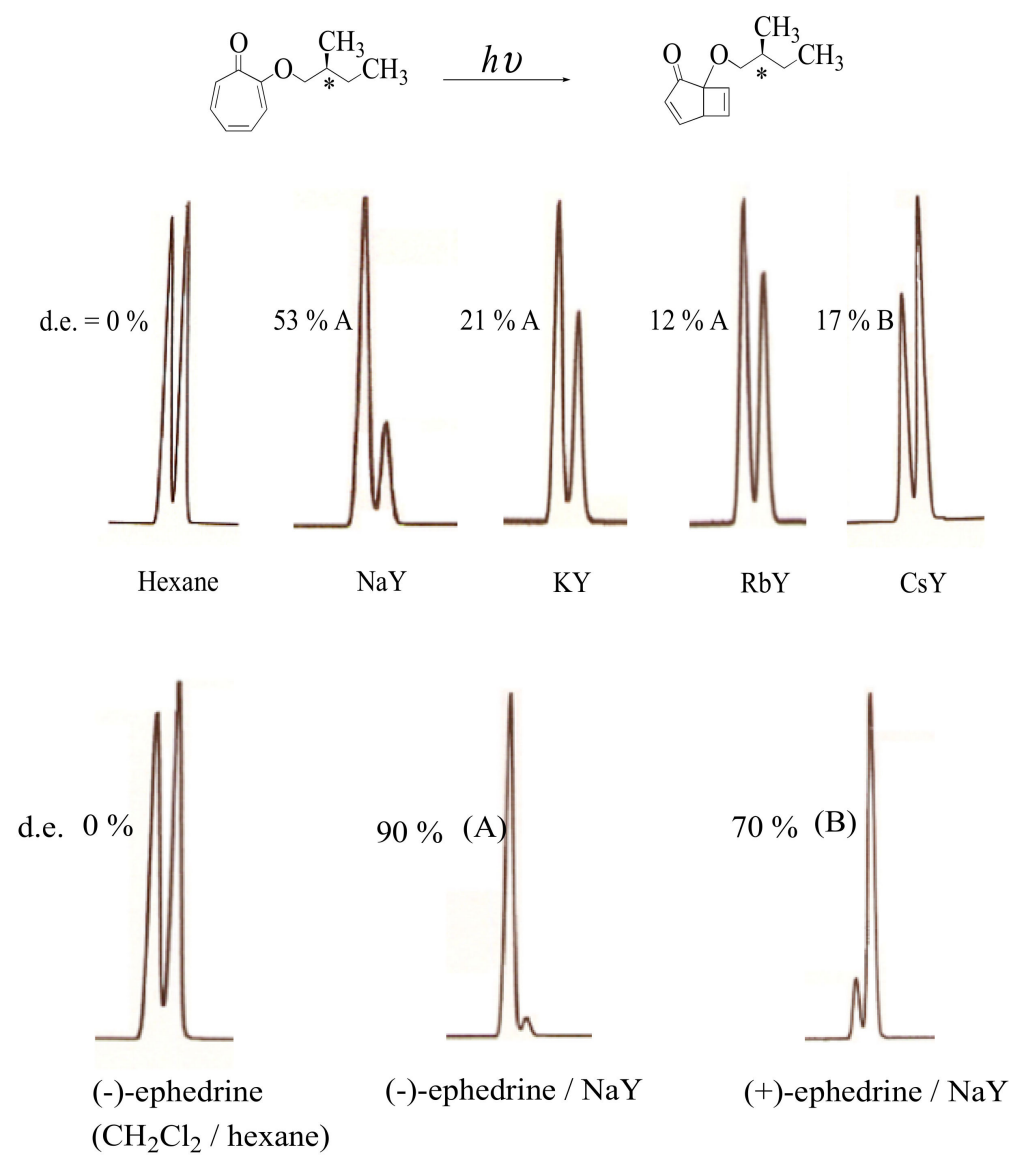
An example of a reaction appended with a remote chiral auxiliary. The effectiveness of the chiral auxiliary is enhanced within chirally-modified zeolites. Top: Chiral induction within achiral zeolites. Effectiveness of chiral auxiliary enhanced within zeolites. Bottom: Chiral induction within chirally-modified zeolites. Diastereomeric excess in the range of 90% from zero in solution is remarkable.

In this review, we highlighted, with examples, the effectiveness of zeolites in bringing about chiral induction on products from achiral reactants. In these chiral inductors, which are not linked to the reactant, achiral reactants and chiral inductors are two independent molecules. Cations within zeolites serve as connectors to bring the chiral inductor and the reactor closer, and thus, to differentiate between the two prochiral faces of the reactant. In addition to these, we have also examined a number of systems where the chiral perturber is covalently linked to the reactant at a remote site. In these systems, the chiral inductor is known as chiral auxiliary. The effectiveness of the chiral auxiliary was more enhanced within zeolites than in solution. One such example, shown in Figure 4, illustrates the power of a zeolite in chiral photochemistry. In this reaction, diastereomeric excess (de) as high as 90% is achieved within zeolites, while in solution the de was zero. For additional examples, please read the listed references [55,60,65,67,70,72,73,74,75,76,77,78,79,80,81,82]. Thus, the value of achiral zeolites in bringing about asymmetric induction in photoreactions of achiral and molecules appended with chiral auxiliaries has been established. The approach described here is likely to gain momentum with the ready availability of the exciting, recently-reported, synthesis of an enantiomerically-enriched zeolite.
